# Ki-67 Expression is a Significant Prognostic Factor Only When Progesterone Receptor Expression is Low in Estrogen Receptor-Positive and HER2-Negative Early Breast Cancer

**DOI:** 10.1155/2019/7386734

**Published:** 2019-12-28

**Authors:** Young-Joon Kang, Han-Byoel Lee, Yun Gyoung Kim, JaiHong Han, Yumi Kim, Tae-Kyung Yoo, Eun-Shin Lee, Hyeong-Gon Moon, Dong-Young Noh, Wonshik Han

**Affiliations:** ^1^Department of Surgery, Incheon St. Mary's Hospital, The Catholic University of Korea, College of Medicine, Incheon, Republic of Korea; ^2^Department of Surgery, Seoul National University College of Medicine, Seoul, Republic of Korea; ^3^Department of Surgery and Cancer Research Institute, Seoul National University College of Medicine, Seoul, Republic of Korea; ^4^Department of Surgery, Bundang Jesaeng General Hospital, Seongnam, Republic of Korea; ^5^Department of Surgery, National Cancer Center, Goyang, Republic of Korea; ^6^Department of Surgery, Seoul St. Mary's Hospital, The Catholic University of Korea, College of Medicine, Seoul, Republic of Korea

## Abstract

**Objective:**

While the value of Ki-67 has been recognized in breast cancer, controversy also exists. The goal of this study is to show the prognostic value of Ki-67 according to progesterone receptor (PgR) expression in patients who have estrogen receptor- (ER-) positive, human epidermal growth factor receptor 2- (HER2-) negative early breast cancer.

**Methods:**

The records of nonmetastatic invasive breast cancer patients who underwent surgery at a single institution between 2009 and 2012 were reviewed. Primary end point was recurrence-free survival (RFS), and secondary end point was overall survival (OS). Ki-67 and PgR were assessed with immunohistochemistry for the tumor after surgery.

**Results:**

A total of 1848 patients were enrolled in this study. 223 (12%) patients had high (≥10%) Ki-67, and 1625 (88%) had low Ki-67 expression. Significantly worse RFS and OS were observed in the high vs. low Ki-67 expression only when the PgR was low (<20%) (*p* < 0.001 and 0.005, respectively, for RFS and OS). There was no significant difference in RFS and OS according to Ki-67 when the PgR was high (*p*=0.120 and 0.076). RFS of four groups according to high/low Ki-67 and PgR expression was compared. The low PgR and high Ki-67 expression group showed worst outcome among them (*p* < 0.001). In a multivariate analysis, high Ki-67 was an independent prognostic factor when the PgR was low (HR 3.05; 95% CI 1.50–6.19; *p*=0.002).

**Conclusions:**

Ki-67 had a value as a prognostic factor only under low PgR expression level in early breast cancer. PgR should be considered in evaluating the prognosis of breast cancer patients using Ki-67.

## 1. Introduction

The prognosis of breast cancer patients is highly variable and depends on several characteristics. Breast cancers represent a heterogeneous group of tumors with histopathological, immunohistochemical (IHC), and genetic differences [1–4]. Clinicohistopathological characteristics have long been used to estimate prognosis and decide on treatment plans. Surrogate approaches that use widely available IHC tests for the estrogen receptor (ER), progesterone receptor (PgR), human epidermal growth factor receptor 2 (HER2), and Ki-67 have since been developed [5, 6]. Recently, genomic information has been integrated into the clinic for predicting breast cancer prognosis and deciding on systemic treatment [7, 8]. However, due to high costs and technical issues, genetic tests may not be available in much of the world. Roles of biomarkers are still important for determining if the patient would benefit from a particular treatment.

Increased proliferation is a hallmark of malignant tumors and is an essential parameter for the prediction of therapy response [7]. Ki-67 is a representative proliferative index. Many studies have used Ki-67 as a presumptive independent predictive marker for treatment with prognostic value for the clinical outcome as well as disease-free and overall survival [9–17]. However, there is also a controversy about the use of Ki-67, with some studies suggesting that Ki-67 lacks prognostic value [18, 19].

The Saint Gallen Consensus recognized a distinction between “luminal A-like” and “luminal B-like” tumor and supported the value of Ki-67 for the robust prognostic information it conveys [20]. The development of multigene analysis has enabled more refined definitions of breast cancer subtypes. Patients with IHC-based luminal A tumors in the low PgR group had significantly poorer disease-free survival than those in the high PgR group [21]. A previous study reported that 51.3% of IHC luminal A tumors with PgR expression ≤20% fell within the intrinsic luminal B classification, and that only 30.9% of IHC luminal B tumors with PgR expression >20% were actually intrinsic luminal B tumors [21]. Thus, the PgR-based IHC classification of luminal subtypes used clinically is somewhat inaccurate, and combining this classification with Ki-67 expression might improve diagnostic accuracy.

The goal of this study was to clarify the independent prognostic value of determining Ki-67 expression. To this end, we investigated the relationship between Ki-67 and PgR expression levels in clinical practice and correlated the expression of these markers with clinicopathologic variables.

## 2. Materials and Methods

### 2.1. Study Population

The records of 1848 patients with pathologically confirmed invasive breast cancer who underwent surgery at the Department of Surgery, Seoul National University Hospital (SNUH; Seoul, South Korea), between July 2009 and December 2012 were retrospectively collected. Patients with ER-positive and HER2-negative breast cancer were included, irrespective of PgR status. Patients diagnosed with in-situ carcinoma or distant metastasis at initial diagnosis or who previously had surgery for breast cancer were excluded, as were those for whom data on PgR or Ki-67 were unavailable. We did not exclude patients who underwent neoadjuvant systemic therapy. IHC analysis was performed from the tissue using core biopsy in a diagnosis. If information from initial tissues was insufficient, we used permanent sections in the patients who were not received neoadjuvant systemic treatment. Biopsy tissue was obtained from patients who underwent neoadjuvant systemic therapy prior to treatment. The study population comprised patients with a diagnosis of stage I to IIIC according to the AJCC (American Joint Committee on Cancer, 8^th^ edition) pathologic staging system. Recurrence was divided into locoregional and distant. Contralateral recurrence was not included among recurrent categories in this study. The primary end point was recurrence-free survival (RFS) in relation to Ki-67 and PgR expression status. The follow-up period corresponded to the interval from surgery to the last date of a hospital visit, regardless of the visited department. The secondary end point was overall survival (OS). For follow-ups, electronic medical records of patients were reviewed up to November 2015; deaths were recorded based on reports as of December 2013. RFS was classified into four groups based on correlations with Ki-67 and PgR expression.

### 2.2. IHC Procedure

ER, PgR, HER2, and Ki-67 expression were determined by IHC in formalin-fixed, paraffin-embedded tissue blocks. Expression levels of hormone receptors, HER2, and Ki-67 were assessed using the avidin-biotin complex technique [22]. Tissues were cut into 4 *μ*m-thick sections, deparaffinized with xylene, rehydrated with a graded ethanol series, and immersed in Tris-buffered saline. Representative sections were immunostained, and more than 10 high-power fields of view were randomly selected and examined under an optical microscope. After antigen retrieval, the sections were incubated with primary antibodies against ER (1DO5; Dako, Denmark; 1 : 50), PgR (PgR636; Dako; 1 : 50), HER2 (CB11; Novocastra Laboratories, upon-Tyne, UK; 1 : 200), and Ki-67 (MIB-1; Dako; 1 : 800) at the indicated dilutions. Sections were then incubated with the biotinylated anti-mouse secondary antibody and stained using streptavidin horseradish peroxidase (Zymed Laboratories, San Francisco, CA, USA). The sections were counterstained with Mayer's hematoxylin, dehydrated, cleared, and then mounted for examination. IHC samples were analyzed by one experienced pathologist at SNUH. The cutoff value used to define low versus high Ki-67 expression was the presence of Ki-67 immunoreactivity in more than 10% of stained nuclei in tumor tissues. The 10% cutoff for Ki-67 was found to have the best predictive value for prognosis at SNUH [23]. Patients were divided into tumors with low (<20%) and high (≥20%) PgR expression. The PgR cutoff of 20% is based on the 2013 Saint Gallen International Breast Cancer Conference [20].

### 2.3. Statistical Analysis

Patients were divided into low and high Ki-67 groups and low and high PgR groups. Clinicopathologic characteristics were assessed using all pairwise comparisons of groups. Categorical variables were compared using chi-square or Fisher's exact test. Student's *t*-test was used for comparing continuous variables between two groups. RFS was defined as the interval from the date of operation to the date of the first observation of a recurrence or the last follow-up date without evidence of recurrence. OS was defined as the interval from the date of operation to the date of death or last follow-up. Survival rates were estimated using the Kaplan–Meier method, and differences between two groups were compared using the log-rank test. In univariate and multivariate analyses of survival rates, Cox proportional hazard regression was used with adjustment for various factors. Cox regression analyses were used to calculate hazard ratios (HRs) and 95% confidence intervals (CIs). Values were two-sided, and statistical significance was defined as a *p* value <0.05. All statistical analyses were performed using SPSS software, version 21 for Windows (IBM Corp., Chicago, IL, USA).

## 3. Results

### 3.1. Clinicopathological Characteristics of Patients

A total of 1,848 patients were enrolled in this study. Clinicopathologic characteristics of analyzed patients were accessed by comparing low and high PgR expression subsets and low and high Ki-67 subsets. The mean age was 49–52 years in each subset. Larger cancers (>2 cm) were more commonly associated with high Ki-67 (*p* < 0.001) and low PgR (*p* < 0.001). Histologic grade (HG) dichotomized samples into low and high subsets, with grade 3 are being classified as high and grade 1 + 2 is classified as low. Node-positive and high-HG samples were also identified in high Ki-67 and low PgR subsets. Significantly, more cases with low PgR underwent neoadjuvant chemotherapy, regardless of Ki-67 status. In addition, more patients with high Ki-67 underwent adjuvant chemotherapy, regardless of PgR status. We also considered tumor characteristics according to the operation method. A total of 719 patients underwent mastectomy, with a larger number of these patients having low PgR expression than high PgR expression. However, there was no significant association between Ki-67 subsets and the frequency of mastectomy. Only PgR subsets were significantly different among axilla surgery types. Recurrence, whether local or distant, was observed in 52 patients. Recurrence was local in 10 cases and distant in 42 cases. Mortality findings are based on public data from the Ministry of the Interior; as of 2013 (last year for which mortality data were examined), 11 patients had died (Table 1).

**Figure 1:**
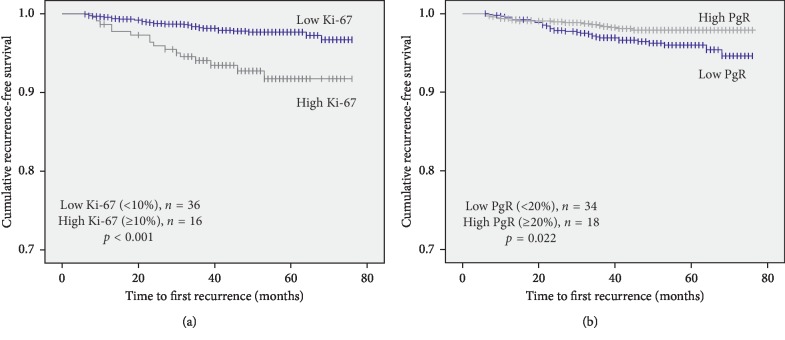
Recurrence-free survival (RFS) according to the Ki-67 index and progesterone receptor expression status. (a) Overall patients (*n* = 1848); RFS according to Ki-67. (b) Overall patients (*n* = 1848); RFS according to progesterone receptor expression.

RFS was significantly better for patients in the low Ki-67 expression group than for those in the high Ki-67 group (*p* < 0.001; Figure 1(a)). RFS was also significantly better for the high PgR expression group than the low PgR expression group (*p*=0.022; Figure 1(b)).

**Figure 2:**
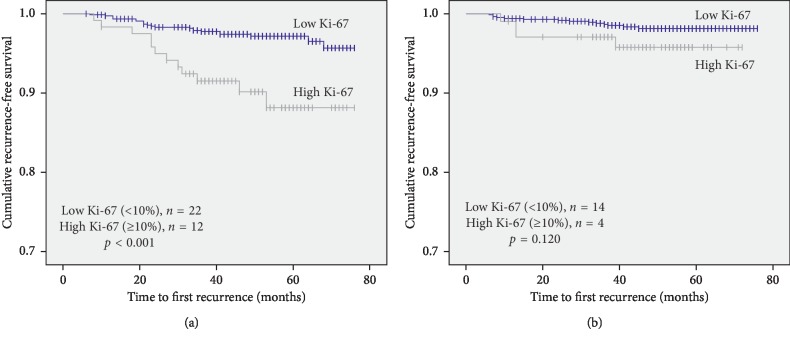
Recurrence-free survival (RFS) of patients in high and low Ki-67 breast cancer according to progesterone receptor expression status. (a) RFS according to Ki-67 in the low progesterone receptor expression subset (PgR < 20%). (b) RFS according to Ki-67 in the high progesterone receptor expression subset (PgR ≥ 20%).

Interestingly, a subset analysis showed that RFS based on Ki-67 expression status was significantly different in the low PgR subset but not in the high PgR subset. Specifically, RFS of patients with high Ki-67 expression and low PgR expression (<20%) was worse than that in the group with low Ki-67 expression and low PgR expression (*p* < 0.001; Figure 2(a)). On the contrary, among patients in the high PgR expression (≥20%) group, there was no significant difference between high and low Ki-67 groups (*p*=0.120; Figure 2(b)).

**Figure 3:**
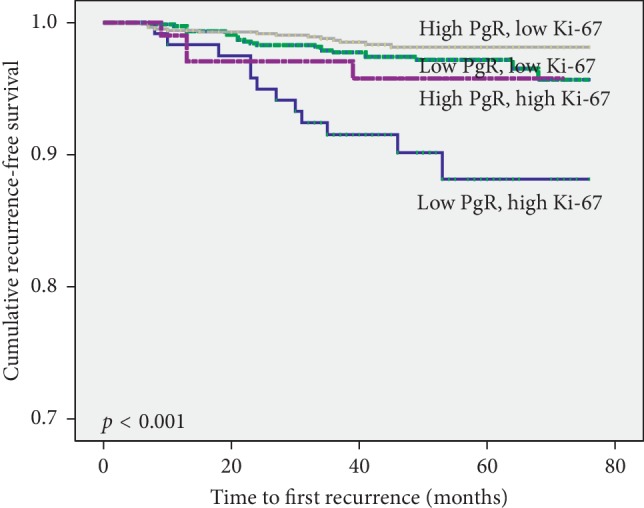
The recurrence-free survival of patients was divided into 4 group combination between Ki-67 and progesterone receptor expression.

RFS was further analyzed by correlating it with subsets divided into four groups based on Ki-67 and PgR expression. Patients with low PgR and high Ki-67 expression showed the poorest outcome compared with the other three groups (*p* < 0.001; Figure 3).

Multivariate Cox regression models showed that Ki-67 was not significantly associated with high PgR expression status, after adjusting for factors including Ki-67 expression group, age, tumor size, nodal status, and HG (HR 2.03; 95% CI 0.61–6.72; *p*=0.247; Table 2). For patients in the low PgR subset, Ki-67 was markedly associated with RFS (HR 3.05; 95% CI 1.50–6.19; *p*=0.002; Table 2). Tumor size was statistically significant with RFS in both PgR status.

**Figure 4:**
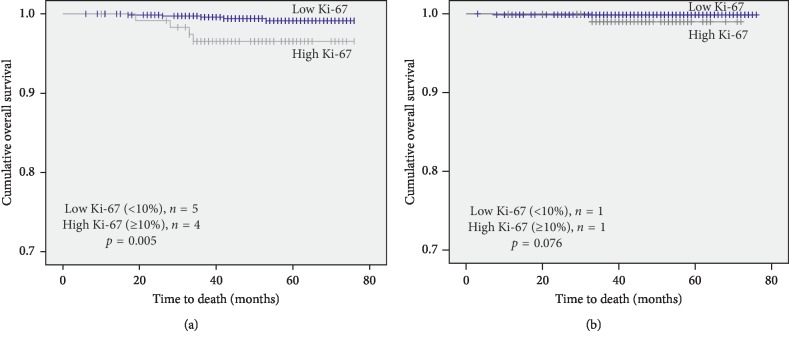
Overall survival (OS) of patients in high and low Ki-67 breast cancer according to progesterone receptor expression status. (a) OS according to Ki-67 in the low progesterone receptor expression subset (PgR < 20%). (b) RFS according to Ki-67 in the high progesterone receptor expression subset (PgR ≥ 20%).

An analysis of the secondary end point, OS, similarly showed superior survival in the low Ki-67 subset with low PgR expression (*p*=0.005; Figure 4(a)) and no significant difference in OS between Ki-67 subsets in the high PgR group (*p*=0.076; Figure 4(b)).

Mean disease-free survival times were 70 months in low PgR/high Ki-67 subset and 75 months in high PgR/low Ki-67 subset. Mean overall survival times were 74 months and 76 months, respectively, for low PgR/high Ki-67 and high PgR/low Ki-67 subset.

## 4. Discussion

Here, we evaluated the value of Ki-67 as an independent prognostic factor for recurrence and survival in nonmetastatic breast cancer patients with ER-positive and HER2-negative tumors. Consistent with previous reports, our study showed that Ki-67 expression exhibited significant prognostic value, but we further demonstrated that Ki-67 is not always an independent prognostic factor. Specifically, Ki-67 had value as a prognostic factor only in the low PgR expression group. In our study, a comparison of RFS among the four expression subgroups revealed the poorest prognosis in the low PgR high Ki-67 subgroup. And the subset was 120 out of 1848 patients. Therefore, active treatment may be considered in about 6% of patients. In terms of predicting prognosis, our findings suggest that combining the Ki-67 expression level with the PgR expression level improves predictive value. Allison et al. reported a strong correlation between high Oncotype DX Recurrence Scores with grade 3 and low-to-absent PR expression and Ki-67 > 10% [24]. In addition, Thakur et al. demonstrated that high Ki-67 status was significantly correlated with the higher Oncotype DX risk-of-recurrence group (low versus high, *p* < 0.001) [25]. If a genomic analysis is not available, the patient of low PgR and high Ki-67 expression in active treatment can be considered in the ER-positive and HER2-negative breast cancer. However, further work is needed, including independent validation and possibly a prospective study, before these findings can be taken towards clinical translation.

Our paper raises several additional issues. Although Ki-67 has been studied as a prognostic marker in breast cancer for more than two decades, there are controversies surrounding the methods used for determining its expression and the overall analytical validity of published results. Analytical validity refers to the ability of a test to produce reproducible and accurate results. For a marker to have prognostic and predictive value, an evidence-based “optimal” cutoff point is essential. Thus, one reason for controversy surrounding the use of Ki-67 as a marker is the absence of a universally accepted standard cutoff value, which has resulted in the use of different specific threshold values by different laboratories [26]. Our institution previously demonstrated that a 10% cutoff value provides the best prognosis-prediction results [23]. This value is different from the cutoff value presented in the Saint Gallen Consensus, which in 2011 defined “low proliferation” tumors as those with a Ki-67 index <14% [5], a cutoff established by comparison with the PAM50 intrinsic multigene molecular test for classification of luminal cancer [27]. During the 2013 Saint Gallen Conference, a majority of panelists voted to raise the threshold indicative of high Ki-67 status to ≥20%. A final definition of a single cutpoint by the Saint Gallen Consensus has remained elusive, owing to the continuous distribution of Ki-67 and analytical and preanalytical barriers to standardized assessment [7]. The cutoff used by our institute is appropriate for our research, but discussions on standardizing Ki-67 assessments to further reduce interobserver variability will continue. It needs to be analyzed to see whether the results reported in terms of the prognostic value of Ki-67 would be recapitulated if 14% or 20% were used instead of 10%. When the cutoff value of Ki-67 was set to 14%, there was no difference according to Ki-67. RFS by Ki-67 had no statistical significance (*p*=0.416; [Supplementary-material supplementary-material-1]) nor showed the difference between the low and high Ki-67 according to the PgR expression subsets (*p*=0.664; [Supplementary-material supplementary-material-1], *p*=0.526; [Supplementary-material supplementary-material-1]). The best strategy is to use Ki-67 as a continuous marker, reflecting the biology of tumor proliferation. Moreover, treatment decision for individual patients should not depend on small differences of Ki-67 around a given cutoff point.

To our knowledge, this is one of the largest retrospective studies to analyze data from a high-quality clinical cancer registry on the routine use and prognostic significance of Ki-67. Although retrospective, it does have the advantage of comprising an unselected, nonmetastatic breast cancer population without selection bias. Notably, pathology and biomarker analyses were prospectively performed in a single, accredited laboratory, and thus represent a ‘real' assessment of the value of IHC in clinical practice.

Despite the various limitations of Ki-67 as a marker, its clinical use in the breast cancer field has been adopted for several reasons. It is used to distinguish luminal tumors and is considered a prognostic factor. Evaluating Ki-67 by IHC is inexpensive and easy to implement without investments in sophisticated equipment, leading to the attractive concept of Ki-67 as a low-cost biomarker. The importance of clinical flexibility is fundamental, given the uncertainties surrounding the tailoring of different chemotherapy options to each case, highlighting the importance of establishing a more accurate role for Ki-67.

In this study, we found that Ki-67 is an effective prognostic marker only in the context of low PgR status. We further found that patients with low PgR and high Ki-67 expression had the worst prognosis in terms of RFS. From a global perspective, genomic signatures will remain difficult to access in the foreseeable future for most patients. Active treatment can be considered as the default in cases of early ER-positive and HER2-negative breast cancer that satisfy conditions of low PgR and high Ki-67.

## 5. Conclusions

Our results show that Ki-67 has prognostic value for recurrence and survival in patients with ER-positive and HER2-negative early breast cancer only in the context of low PgR expression level. Thus, PgR expression should also be considered in evaluating the prognosis of breast cancer patients using Ki-67.

## Figures and Tables

**Table 1:** Clinical and histopathologic characteristics.

Characteristics		Study population (*n* = 1848)					
		PgR low (*n* = 895)	PgR high (*n* = 953)	*p* value	Ki-67 low (*n* = 1625)	Ki-67 high (*n* = 223)	*p* value
Age, mean (range)		52.2 (20–81)	49.2 (22–85)	<0.001	50.9 (22–82)	48.6 (20–85)	<0.001
Menopausal status				<0.001			0.191
Premenopausal		413 (46.2)	604 (63.4)		884 (54.4)	133 (59.6)	
Postmenopausal		463 (51.7)	324 (34.0)		704 (43.3)	83 (37.2)	
Unknown		19 (2.1)	25 (2.6)		37 (2.3)	7 (3.1)	
Histologic type				0.147			0.183
IDCa		830 (92.7)	860 (90.2)		1479 (91.0)	211 (94.6)	
ILCa		30 (3.4)	46 (4.8)		71 (4.4)	5 (2.2)	
Others		35 (3.9)	47 (4.9)		75 (4.6)	7 (3.1)	
Tumor size (cm)				<0.001			<0.001
≤2		452 (50.5)	564 (59.2)		919 (56.6)	97 (43.5)	
>2		443 (49.5)	389 (40.8)		706 (43.4)	126 (56.5)	
LN metastasis				<0.001			0.007
Negative		541 (60.4)	693 (72.7)		1103 (67.9)	131 (58.7)	
Positive		354 (39.6)	260 (27.3)		522 (32.1)	92 (41.3)	
Histologic grade				<0.001			<0.001
Low (I, II)		565 (66.6)	727 (76.3)		1243 (76.5)	80 (35.9)	
High (III)		299 (33.4)	226 (23.7)		382 (23.5)	143 (64.1)	
Breast operation				0.001			0.443
Partial mastectomy		511 (57.1)	618 (64.8)		998 (61.4)	131 (58.7)	
Total mastectomy		384 (42.9)	335 (35.2)		627 (38.6)	92 (41.3)	
Axilla operation				<0.001			0.290
Sentinel node biopsy		491 (54.9)	643 (67.5)		1005 (61.8)	129 (57.8)	
Axilla node dissection		404 (45.1)	303 (31.8)		613 (37.7)	94 (42.2)	
Not done		0 (0.0)	7 (0.7)		7 (0.4)	0 (0.0)	
Recurrence				0.043			<0.001
No		861 (96.2)	935 (98.1)		1589 (97.8)	207 (92.8)	
Locoregional		6 (0.7)	4 (0.4)		7 (0.4)	3 (1.3)	
Distant		28 (3.1)	14 (1.5)		29 (1.8)	13 (5.8)	
Death				0.022			0.009
No		885 (98.9)	951 (99.8)		1618 (99.6)	218 (97.8)	
Yes		9 (1.0)	2 (0.2)		6 (0.4)	5 (2.2)	
Unknown		1 (0.1)	0 (0.0)		1 (0.1)	0 (0.0)	

PgR: progesterone receptor, IDCa: invasive ductal carcinoma, ILCa: invasive lobular carcinoma, and LN: lymph node. Patient outcomes according to Ki-67 and PgR expression.

**Table 2:** Multivariate Cox regression analysis for recurrence-free survival.

		Low PgR status		High PgR status	
		HR (95% CI)	*p*	HR (95% CI)	*p*
Age		1.00 (0.97–1.03)	0.856	0.99 (0.95–1.04)	0.713
Tumor size	≥2 cm, <2 cm	7.20 (2.53–20.51)	<0.001	5.40 (1.49–19.60)	0.010
Nodal status	Yes, no	1.66 (0.79–3.45)	0.179	2.53 (0.94–6.77)	0.065
Ki-67	≥10%, <10%	3.05 (1.50–6.19)	0.002	2.03 (0.61–6.72)	0.247
HG	High, low	1.78 (0.85–3.72)	0.127	0.82 (0.28–2.40)	0.719

## Data Availability

The demographic and clinical data collected for the purpose of the statistical analysis to support the findings of this study are available from the corresponding author upon request.

## Supplementary Materials

Supplementary Materials Appendix 1: recurrence-free survival (RFS) according to the Ki-67 index by a 14% cutoff value. Appendix 2a: recurrence-free survival (RFS) according to Ki-67 (14% cutoff value) in the low progesterone receptor subset. Appendix 2b: RFS according to Ki-67 (14% cutoff value) in the high progesterone receptor subset.

## References

[1] van de Vijver M. J., He Y. D., van’t Veer L. J. (2002). A gene-expression signature as a predictor of survival in breast cancer. *New England Journal of Medicine*.

[2] Parker J. S., Mullins M., Cheang M. C. U. (2009). Supervised risk predictor of breast cancer based on intrinsic subtypes. *Journal of Clinical Oncology*.

[3] Sorlie T., Perou C. M., Tibshirani R. (2001). Gene expression patterns of breast carcinomas distinguish tumor subclasses with clinical implications. *Proceedings of the National Academy of Sciences*.

[4] Paik S., Shak S., Tang G. (2004). A multigene assay to predict recurrence of tamoxifen-treated, node-negative breast cancer. *New England Journal of Medicine*.

[5] Goldhirsch A., Wood W. C., Coates A. S. (2011). Strategies for subtypes-dealing with the diversity of breast cancer: highlights of the st gallen international expert CONSENSUS on the primary therapy of early breast cancer 2011. *Annals of Oncology*.

[6] Nielsen T. O., Hsu F. D., Jensen K. (2004). Immunohistochemical and clinical characterization of the basal-like subtype of invasive breast carcinoma. *Clinical Cancer Research*.

[7] Coates A. S., Winer E. P., Goldhirsch A. (2015). Tailoring therapies-improving the management of early breast cancer: st gallen international expert Consensus on the primary therapy of early breast cancer 2015. *Annals of Oncology*.

[8] NCCN Clinical Practice Guidelines in Oncology for Breast Cancer Version 1, 2017, http://wwwnccnorg/professionals/physician_gls/f_guidelinesasp.2017

[9] Esposito A., Criscitiello C., Curigliano G. (2015). Highlights from the 14(th) St Gallen International Breast Cancer Conference 2015 in Vienna: dealing with classification, prognostication, and prediction refinement to personalize the treatment of patients with early breast cancer. *Ecancermedicalscience*.

[10] Chang J., Ormerod M., Powles T. J., Allred D. C., Ashley S. E., Dowsett M. (2000). Apoptosis and proliferation as predictors of chemotherapy response in patients with breast carcinoma. *Cancer*.

[11] Faneyte I. F., Schrama J. G., Peterse J. L., Remijnse P. L., Rodenhuis S., van de Vijver M. J. (2003). Breast cancer response to neoadjuvant chemotherapy: predictive markers and relation with outcome. *British Journal of Cancer*.

[12] Colozza M., Azambuja E., Cardoso F., Sotiriou C., Larsimont D., Piccart M. J. (2005). Proliferative markers as prognostic and predictive tools in early breast cancer: where are we now?. *Annals of Oncology*.

[13] Tamaki K., Moriya T., Sato Y. (2009). Vasohibin-1 in human breast carcinoma: a potential negative feedback regulator of angiogenesis. *Cancer Science*.

[14] Trihia H., Murray S., Price K. (2003). Ki-67 expression in breast carcinoma. *Cancer*.

[15] Petrelli F., Viale G., Cabiddu M., Barni S. (2015). Prognostic value of different cut-off levels of Ki-67 in breast cancer: a systematic review and meta-analysis of 64,196 patients. *Breast Cancer Research and Treatment*.

[16] de Azambuja E., Cardoso F., de Castro G. (2007). Ki-67 as prognostic marker in early breast cancer: a meta-analysis of published studies involving 12 155 patients. *British Journal of Cancer*.

[17] Luporsi E., André F., Spyratos F. (2012). Ki-67: level of evidence and methodological considerations for its role in the clinical management of breast cancer: analytical and critical review. *Breast Cancer Research and Treatment*.

[18] Nielsen T. O., Parker J. S., Leung S. (2010). A comparison of PAM50 intrinsic subtyping with immunohistochemistry and clinical prognostic factors in tamoxifen-treated estrogen receptor-positive breast cancer. *Clinical Cancer Research*.

[19] Harris L., Fritsche H., Mennel R. (2007). American Society of Clinical Oncology 2007 update of recommendations for the use of tumor markers in breast cancer. *Journal of Clinical Oncology*.

[20] Goldhirsch A., Winer E. P., Coates A. S. (2013). Personalizing the treatment of women with early breast cancer: highlights of the st gallen international expert consensus on the primary therapy of early breast cancer 2013. *Annals of Oncology: Official Journal of the European Society for Medical Oncology*.

[21] Prat A., Cheang M. C. U., Martín M. (2013). Prognostic significance of progesterone receptor-positive tumor cells within immunohistochemically defined luminal A breast cancer. *Journal of Clinical Oncology*.

[22] Tiezzi D. G., Andrade J. M., Ribeiro-Silva A., Zola F. E., Marana H. R., Tiezzi M. G. (2007). HER-2, p53, p21 and hormonal receptors proteins expression as predictive factors of response and prognosis in locally advanced breast cancer treated with neoadjuvant docetaxel plus epirubicin combination. *BMC Cancer*.

[23] Jung S.-Y., Han W., Lee J. W. (2009). Ki-67 expression gives additional prognostic information on St. Gallen 2007 and Adjuvant! Online risk categories in early breast cancer. *Annals of Surgical Oncology*.

[24] Allison K. H., Kandalaft P. L., Sitlani C. M., Dintzis S. M., Gown A. M. (2012). Routine pathologic parameters can predict oncotype DXTM recurrence scores in subsets of ER positive patients: who does not always need testing?. *Breast Cancer Research and Treatment*.

[25] Thakur S. S., Li H., Chan A. M. Y. (2018). The use of automated Ki67 analysis to predict oncotype DX risk-of-recurrence categories in early-stage breast cancer. *PLoS One*.

[26] Polley M.-Y. C., Leung S. C. Y., Gao D. (2015). An international study to increase concordance in Ki67 scoring. *Modern Pathology*.

[27] Cheang M. C. U., Chia S. K., Voduc D. (2009). Ki67 index, HER2 status, and prognosis of patients with luminal B breast cancer. *JNCI: Journal of the National Cancer Institute*.

