# Supplementary material for: Ki-67 Expression is a Significant Prognostic Factor Only When Progesterone Receptor Expression is Low in Estrogen Receptor-Positive and HER2-Negative Early Breast Cancer
Source: J Oncol. 2019 Dec 28;2019:7386734. doi: 10.1155/2019/7386734 (PMC6949686; doi:10.1155/2019/7386734)
Supplement: Supplementary Materials — Appendix 1: recurrence-free survival (RFS) according to the Ki-67 index by a 14% cutoff value. Appendix 2a: recurrence-free survival (RFS) according to Ki-67 (14% cutoff value) in the low progesterone receptor subset. Appendix 2b: RFS according to Ki-67 (14% cutoff value) in the high progesterone receptor subset. [file 7386734.f1.pdf]

Appendix 1

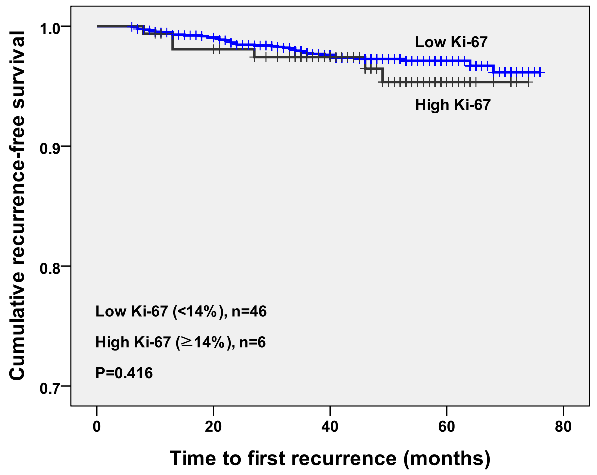

Recurrence free survival (RFS) according to Ki-67 index by 14% cut-off value

Appendix 2

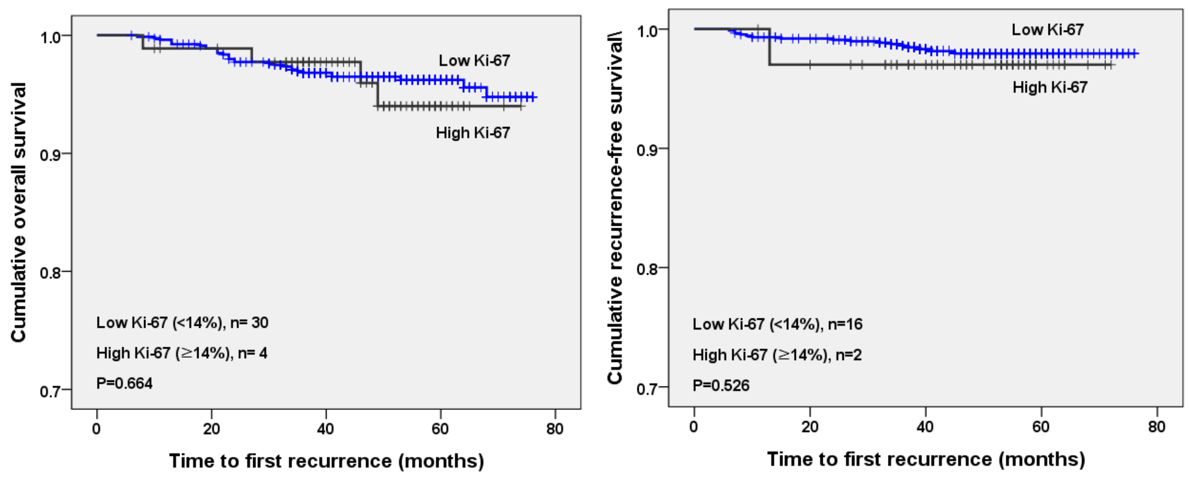

a. RFS in the low PgR subset b. RFS in the high PgR subset

RFS of patients in high and low Ki-67 by 14% cut-off value according to progesterone receptor expression status (a) RFS according to Ki-67 in the low PgR subset (b) RFS according to Ki-67 in the high PgR subset
